# Risks of stillbirth and neonatal death with advancing gestation at term: A systematic review and meta-analysis of cohort studies of 15 million pregnancies

**DOI:** 10.1371/journal.pmed.1002838

**Published:** 2019-07-02

**Authors:** Javaid Muglu, Henna Rather, David Arroyo-Manzano, Sohinee Bhattacharya, Imelda Balchin, Asma Khalil, Basky Thilaganathan, Khalid S. Khan, Javier Zamora, Shakila Thangaratinam

**Affiliations:** 1 Women’s and Sexual Health Division, University Hospital Lewisham, Lewisham and Greenwich NHS Trust, London, United Kingdom; 2 Women’s Division, North Middlesex University Hospital, London, United Kingdom; 3 Clinical Biostatistics Unit, Hospital Ramon y Cajal (IRYCIS) and CIBER Epidemiology and Public Health (CIBERESP), Madrid, Spain; 4 Dugald Baird Centre for Research on Women’s Health, Aberdeen Maternity Hospital, University of Aberdeen, Aberdeen, United Kingdom; 5 University of Malaya, Kuala Lumpur, Malaysia; 6 Fetal Medicine Unit, St George’s University Hospitals NHS Foundation Trust, London, United Kingdom; 7 Molecular and Clinical Sciences Research Institute, St George’s University of London, London, United Kingdom; 8 Barts Research Centre for Women’s Health, Women’s Health Research Unit, Barts and The London School of Medicine and Dentistry, Queen Mary University of London, London, United Kingdom; 9 Multidisciplinary Evidence Synthesis Hub, Barts and The London School of Medicine and Dentistry, Queen Mary University of London, London, United Kingdom; Cambridge University, UNITED KINGDOM

## Abstract

**Background:**

Despite advances in healthcare, stillbirth rates remain relatively unchanged. We conducted a systematic review to quantify the risks of stillbirth and neonatal death at term (from 37 weeks gestation) according to gestational age.

**Methods and findings:**

We searched the major electronic databases Medline, Embase, and Google Scholar (January 1990–October 2018) without language restrictions. We included cohort studies on term pregnancies that provided estimates of stillbirths or neonatal deaths by gestation week. We estimated the additional weekly risk of stillbirth in term pregnancies that continued versus delivered at various gestational ages. We compared week-specific neonatal mortality rates by gestational age at delivery. We used mixed-effects logistic regression models with random intercepts, and computed risk ratios (RRs), odds ratios (ORs), and 95% confidence intervals (CIs). Thirteen studies (15 million pregnancies, 17,830 stillbirths) were included. All studies were from high-income countries. Four studies provided the risks of stillbirth in mothers of White and Black race, 2 in mothers of White and Asian race, 5 in mothers of White race only, and 2 in mothers of Black race only. The prospective risk of stillbirth increased with gestational age from 0.11 per 1,000 pregnancies at 37 weeks (95% CI 0.07 to 0.15) to 3.18 per 1,000 at 42 weeks (95% CI 1.84 to 4.35). Neonatal mortality increased when pregnancies continued beyond 41 weeks; the risk increased significantly for deliveries at 42 versus 41 weeks gestation (RR 1.87, 95% CI 1.07 to 2.86, *p* = 0.012). One additional stillbirth occurred for every 1,449 (95% CI 1,237 to 1,747) pregnancies that advanced from 40 to 41 weeks. Limitations include variations in the definition of low-risk pregnancy, the wide time span of the studies, the use of registry-based data, and potential confounders affecting the outcome.

**Conclusions:**

Our findings suggest there is a significant additional risk of stillbirth, with no corresponding reduction in neonatal mortality, when term pregnancies continue to 41 weeks compared to delivery at 40 weeks.

**Systematic review registration:**

PROSPERO CRD42015013785

## Abstract 2

Javaid Muglu and colleagues reveal the increasing risk of stillbirths and neontatal deaths for pregnancies that go beyond 41 weeks.

## Author summary

**Why was this study done?:**

- A third of stillborn babies in the UK are born at term (>37 weeks) and were previously considered to be healthy.
- Prolongation of pregnancy at term is a known risk factor for stillbirth. Currently women are routinely offered induction of labour after 41 weeks gestation to avoid stillbirth. But 1 in 3 women have a stillborn baby prior to this gestational age.
- Mothers need robust estimates of potential risks of stillbirth and newborn death at term to make decisions on timing of delivery.

**What did the researchers do and find?:**

- We collated data from all relevant studies found in a systematic review and determined the additional risks of stillbirth and newborn death in mothers at term gestation, by comparing pregnancies that continued versus delivered at various gestational ages,.
- We found a steady increase in the risk of stillbirth with advancing gestation at term. In mothers who continued their pregnancy to 41 weeks, there was a 64% increase in the risk of stillbirth compared to those who delivered at 40 weeks, with 1 additional mother having a stillborn baby for every 1,449 women.
- The risks of newborn death remained constant between 38 and 41 weeks, and only increased beyond 41 weeks.

**What do these findings mean?:**

- Any mother considering prolongation of pregnancy beyond 37 weeks should be informed of the additional small but significantly increased risk of stillbirth with advancing gestation.
- Women planning delivery before 41 weeks gestation can be reassured that there is no additional risk of newborn death when delivering between 38 and 41 weeks.
- Our findings are limited by variations in the definition of low-risk pregnancy, the inclusion of studies over a long period of time, and the possibility of other unaccounted factors that may have affected the outcomes.

## Introduction

Despite advances in antenatal and intrapartum care, stillbirth continues to be a major burden [1]. More than 3,000 babies are stillborn every year in the UK—with a third of them considered to be apparently healthy term infants (37 weeks gestation or beyond) [2]. Stillbirth at term in an otherwise low-risk pregnancy [3,4] devastates parents with its unexpectedness. The UK’s recent Maternity Safety Strategy initiative aims to halve the stillbirth rate by 2025 [5]. Such an effort requires an understanding of the magnitude of the problem through collation of large datasets, particularly for key factors like gestational age, for which existing information is imprecise [6].

Prolonged pregnancy is a known risk factor for stillbirth. To avoid this adverse outcome, women are routinely offered induction of labour after 41 weeks gestation [6–8]. This recommendation is based on evidence of increased stillbirth risk beyond 41 weeks [9]. However, 1 in 3 stillbirths occur prior to 41 weeks gestation [2–4]. The stillbirth risks before 41 weeks are not routinely discussed with women who have no clinical indication for delivery. This is in part because of how ‘term pregnancy’ is defined as normal in standard texts [10], and in part because of concerns about adverse neonatal outcomes that may occur from delivery before 41 weeks [11].

Individual studies on the risk of stillbirth in what is considered as normal term gestation vary in the magnitude and consistency of findings by gestational week [12–14]. Corresponding neonatal mortality estimates are imprecise [13,15,16]. We undertook a systematic review to evaluate the additional weekly risks of stillbirth in term pregnancies that continue versus deliver at various gestational ages. We also assessed the week-specific risks of neonatal death by gestational age at birth.

## Methods

We undertook the review using a prospectively registered protocol (PROSPERO CRD42015013785) and report our findings in line with Preferred Reporting Items for Systematic Reviews and Meta-Analyses (PRISMA) recommendations [17]. Ethics approval was not needed.

### Literature search and study identification

We searched Medline, Embase, and Google Scholar from January 1990 to March 2017 for studies reporting rates of stillbirth and/or neonatal death at various gestational ages in apparently low-risk term pregnancies, and updated the search again to October 2018. We used the following search terms for the population: ‘term pregnancy’, ‘prolonged pregnancy’, ‘post term’, and ‘postdates’; these were combined with terms relevant to the outcomes such as ‘stillbirth’, ‘intrauterine death’, ‘fetal death’, ‘perinatal death’, and ‘perinatal mortality’. We undertook a separate search for studies reporting only neonatal death using the terms ‘neonatal mortality’, ‘newborn death’, and ‘neonatal death’ and combined these with ‘term pregnancy’, ‘singleton’, and ‘low-risk pregnancy’ (S1 Appendix). There were no language restrictions. We manually searched the reference lists of relevant studies for more relevant data, and contacted the authors and researchers in the field for additional studies or relevant information where required.

### Study selection

We selected the studies in a 2-stage process. First 2 independent reviewers (JM and HR) screened the titles and abstracts to identify eligible studies, and then they retrieved the relevant full texts for detailed assessment. Any disagreements on the eligibility of the studies were resolved with a third reviewer (ST). We included cohort studies (including those nested within randomised trials) on pregnant women at term gestation without a prespecified indication for early delivery, if they provided weekly estimates of stillbirths. We excluded abstracts, letters, case reports, case series, and animal studies, and studies that only included women with pre-existing medical conditions, congenital fetal malformations, complications such as preeclampsia, gestational diabetes, or small-for-gestational-age fetuses, or women who needed planned delivery before 37 weeks for maternal or fetal reasons. We defined term pregnancies as pregnancies with a gestational age of 37 completed weeks or beyond [18]. Stillbirth was defined as the death of a baby before birth, which included both antenatal and intrapartum deaths [19]. Any newborn death before 28 days of age was classed as a neonatal death [20]. We defined a low-risk pregnancy as that in which a healthy woman with apparently uncomplicated pregnancy enters labour with a low risk of developing intrapartum complications [21].

### Quality assessment and data extraction

Two independent reviewers (JM and HR) assessed the quality of the individual studies, both for internal (risk of bias) and external (the representativeness of the population) validity [22]. For internal validity, we studied the individual features of the study such as the design, method of sampling, ascertainment of the outcome, appropriate determination of gestational age, and adequacy of follow-up [23]. We considered studies with a prospective design, random or consecutive sampling, use of first-trimester ultrasound to determine gestational age [24], and follow-up rates of over 80% to have a low risk of bias. For external validity, we considered a population to be clearly defined as representative of low-risk pregnancy if it met the following criteria: a clear definition of low-risk pregnancy, exclusion of pregnancies with congenital fetal malformations, and exclusion of multiple pregnancies. Any discrepancies were resolved after discussion with a third reviewer (ST). Data were extracted in duplicate by 2 reviewers (JM and HR). We extracted the number of ongoing pregnancies, number of deliveries, and number of events (stillbirths or neonatal deaths) per week.

### Analysis

In the first step, for each study we calculated the gestation-week-specific prospective risk of stillbirth from the number of stillbirths that occurred in that week divided by the number of pregnancies that were considered to be at risk. The ‘at risk’ pregnancies were determined from the number of women who were still pregnant at the beginning of the week minus half the number who delivered that week [23]. We obtained pooled week-specific risks by using a multilevel (studies and women) mixed-effects logistic regression model without covariates and with random intercepts [25]. The overall week-specific rates of neonatal death were calculated using the same model: The number of neonatal deaths that occurred in a particular week were divided by the number of deliveries in that period.

In the next step, we compared the change in overall week-specific risk of the event (either stillbirth or neonatal death) between 2 consecutive weeks by calculating the risk ratio (RR). We calculated the RR by dividing week-specific risks that were obtained after fitting the corresponding logistic models. Non-parametric 95% confidence intervals (CIs) were obtained after fitting the logistic models on each of the 1,000 bootstrap samples (not stratified by study). After calculating the distribution of RRs for all gestational ages, we chose the 2.5th and 97.5th percentiles to represent the non-parametric limits of the 95% CI. For each gestational week, we also estimated the number of pregnancies at risk, i.e., the number of pregnancies that if continued to the next week will experience 1 additional stillbirth, compared to delivery at that gestational week [26,27].

We planned subgroup and sensitivity analyses a priori to determine whether the risks of stillbirth and neonatal death at term varied according to maternal characteristics such as race (White, Black, Asian, Other), body mass index (normal, overweight, obese), and age; study characteristics such as quality (risk of bias), country income status (low, middle, high), and time period; or restriction of assessments to those studies that excluded fetuses with congenital malformations and studies that used a strict definition (criterion) of low-risk pregnancy. For subgroup analysis, we compared the week-specific risks of stillbirth in women of Black versus White race by including race as a covariate in the logistic model, with White race as reference. We reported the estimates as odds ratios (ORs) with 95% CIs for various gestational ages.

We estimated the heterogeneity using the tau-squared statistic, with a value of 0 indicating no between-study variance. Publication bias and small study effect were assessed with funnel plots representing weekly event rate (logit scale) versus its standard error. Begg’s and Egger’s tests were used to determine funnel asymmetry [28,29]. All analyses were carried out in Stata version 13.1.

## Results

**Fig 1:**
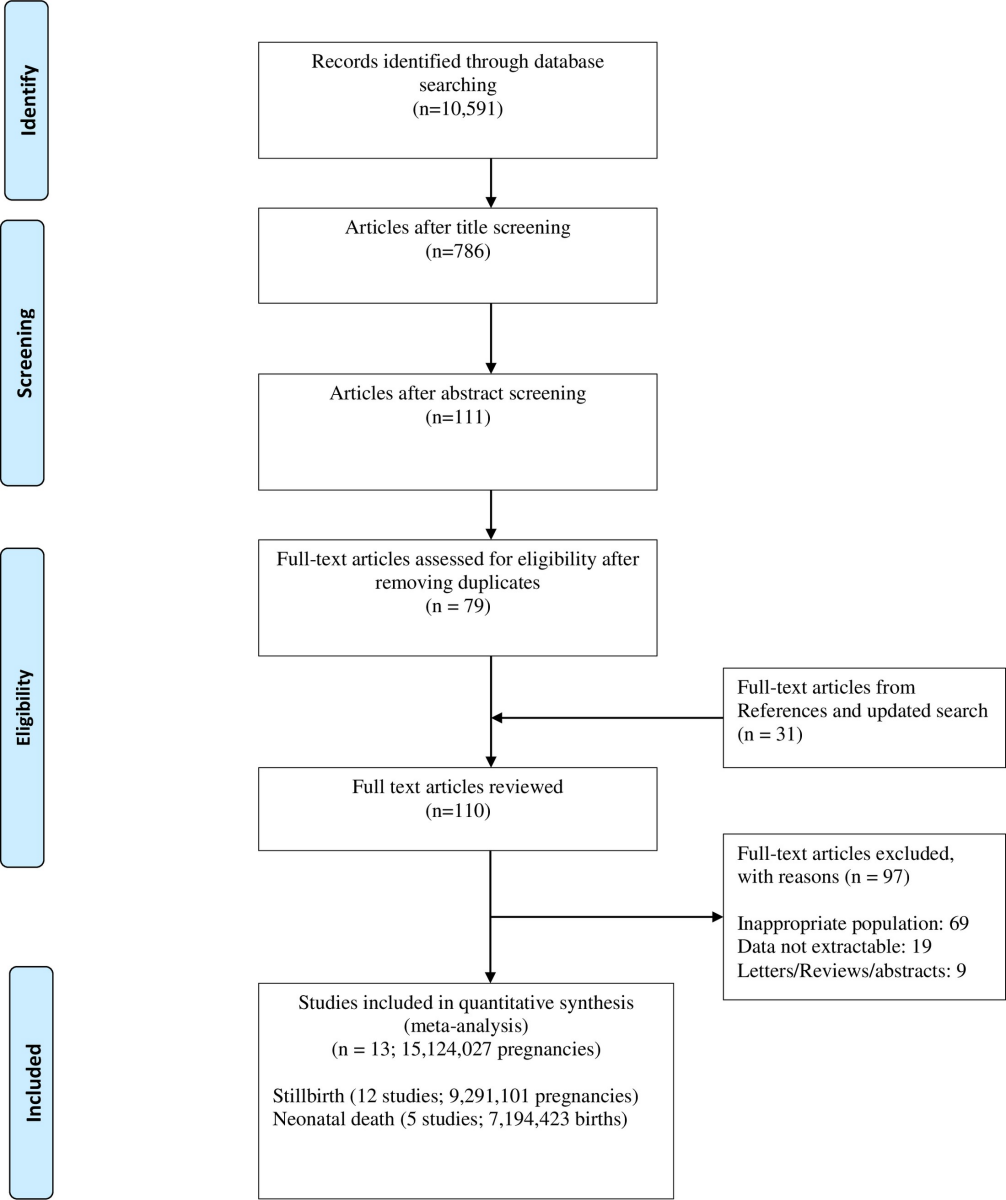
Flow diagram of study selection in systematic review of prospective risk of stillbirth and neonatal death in pregnancies continued to term.

From 10,591 citations, we included 13 studies (15,124,027 pregnancies), which reported 17,830 stillbirths and 2,348 neonatal deaths (Fig 1).

### Characteristics of the included studies

Ten of the 13 studies included only singleton pregnancies [12,14,30–35], 6 studies excluded pregnancies complicated by congenital fetal malformations [14,32–35], and 4 included women without any medical complications [30,35]. Twelve studies provided weekly rates of stillbirth only [12–14,30–35], 1 provided rates of neonatal death only [36], and 4 provided rates of both stillbirth and neonatal death [13,30,34]. Four studies provided data to compare the weekly risks of stillbirth for women of White versus Black race [12,31,33], and 2 for White versus Asian race [33]. There were no major differences between the studies in the definitions of stillbirth and neonatal mortality. Ten studies provided clear definitions of stillbirth and neonatal death [13,14,30,31–37]. Three studies used registry entry data on stillbirth and neonatal death for analysis (Table 1).

**Table 1:** Characteristics of individual studies included in systematic review and meta-analysis of stillbirths and neonatal deaths in pregnancies continued to term.

Study [reference] (country)	Study type/quality	Inclusion	Exclusion	Number in study	Definition of GA	Outcomes
Balchin 2007 [30] (UK)	Prospective cohort, in 15 maternity units from 1988–2000	Nulliparous White, Asian, or Black women delivering singleton weighing at least 500 g at 24–43 weeks	Preterm birth, multiple birth, previous poor obstetric history; we excluded data below 37 weeks gestation	476,371	LMP/USS (weeks) (BPD)	Perinatal mortality, stillbirths, neonatal deaths
Ferguson 1990 [12] (US)	Retrospective cohort in Illinois from 1980–1984	Singleton birth at 25–42 weeks	We excluded data below 37 weeks gestation	711,195	NS	Stillbirths (fetal deaths)
Feldman 1992 [37] (US)	Retrospective cohort from birth records of New York City Department of Health from 1987–1989	Singleton and multiple births at 26–42 weeks	We excluded data below 37 weeks gestation	328,864	LMP (weeks)	Stillbirths
Ferguson 1994 [31] (US)	Retrospective cohort in Illinois from 1984–1988	Singleton births at 25–42 weeks; data reporting birth weight, GA, and White or Black race	We excluded data below 37 weeks gestation	669,491	LMP (weeks)	Stillbirths (fetal deaths)
Hilder 1998 [13] (UK)	Retrospective cohort from notified births in 18 hospitals in London from 1989–1991	Singleton and multiple births at 37–43 weeks	We excluded data below 37 weeks gestation	158,171	LMP/USS (weeks)	Still births, neonatal deaths
Hedegaard 2014 [35] (Denmark)	Retrospective cohort from Danish birth register from 2000–2012	Singleton and multiple births (twin counted as 2 pregnancies and 2 births) at 37–42+ weeks	—	772,483	USS (LMP) (weeks)	Stillbirths
Khalil 2015 (unpublished) (UK)	Retrospective cohort from St George’s Hospital from 2000–2015	Singleton pregnancies at 37–43 weeks; raw data provided by author	Multiple pregnancies, pregnancies with medical problems, congenital malformations	91,693	USS (weeks)	Stillbirths neonatal deaths
Nakling 2006 [32] (Norway)	Prospective study in 1 Norwegian county from 1989–1999	Singleton births at 37–42+ weeks	Multiple births, lack of USS information, delivery before 37 weeks, congenital abnormalities	17,493	USS (weeks)	Stillbirths
Rasmussen 2003 [14] (Norway)	Retrospective cohort from records of births in Norway from 1967–1998	Singleton births at 28–43+ weeks; raw data provided by the author	Multiple births, congenital anomalies, lack of information about LMP, GA < 28 weeks; we excluded data below 37 weeks gestation	1,595,535	LMP (weeks)	Stillbirths
Rosenstein 2012 [33] (US)	Retrospective cohort study including term births in California from 1997–2006	Singleton pregnancies at 37–42 weeks; raw data provided by the author	Multiple births, DM, HTN, congenital abnormality, lack of information on LMP	3,759,300	LMP (weeks)	Stillbirths, infant deaths
Smith 2001 [34] (UK)	Retrospective cohort study including term births in Scotland from 1985–1996	Singleton pregnancies at term (37–43 weeks)	Multiple births, congenital abnormalities, >43 weeks gestation	700,878	LMP/USS (weeks)	Stillbirths, neonatal deaths
Zhang 2009 [36] (US)	Retrospective cohort in US from 1995–2001	Singleton live births at 37–41 weeks from National Center for Health Statistics; low-risk data provided by author; spontaneous (non-induced) vaginal births with no medical problems	Births with known congenital abnormalities	5,768,536	LMP (weeks)	Neonatal deaths, post-neonatal deaths
Bhattacharya 2015 (unpublished) (UK)	Retrospective cohort in Scotland from 2002–2012	Singleton pregnancies at 37–43 weeks; raw data provided by author	Multiple pregnancies, PET, GDM, APH	9,627	NS	Stillbirths

APH, antepartum haemorrhage; BP, biparietal diameter; DM, diabetes mellitus; GA, gestational age; GDM, gestational diabetes; HTN, hypertension; LMP, last menstrual period; NS, not specified; PET, preeclampsia.

### Quality of the included studies

**Fig 2:**
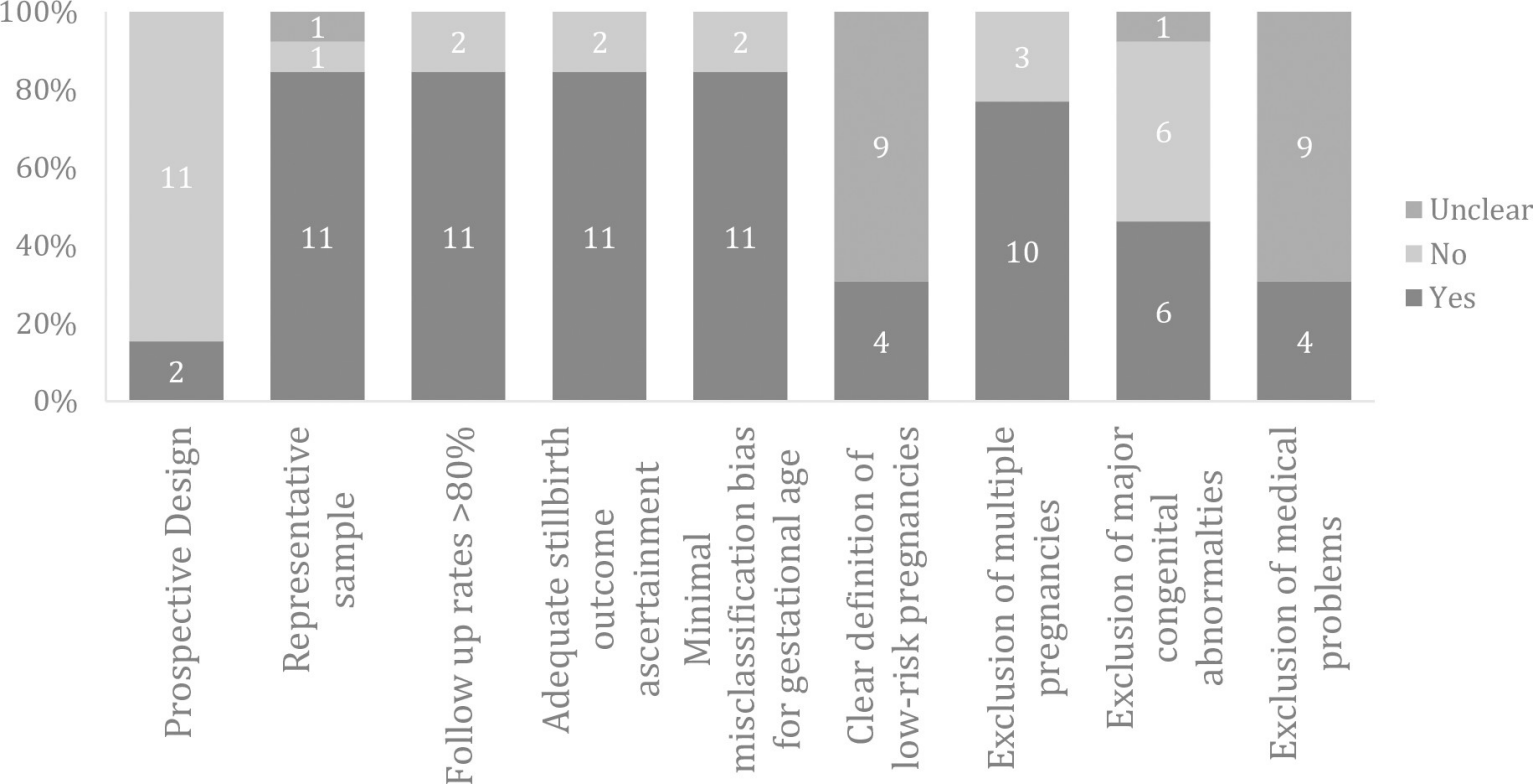
Risk of bias in studies included in the systematic review on prospective risk of stillbirth and neonatal death in pregnancies continued to term.

Eleven studies (11/13; 85%) were retrospective analyses of prospectively gathered datasets. Most studies used consecutive sampling (12/13, 92%), achieved adequate follow-up (11/13; 85%), and had low ascertainment bias for determining the stillbirth outcome (11/13; 85%) and low misclassification bias for assessing the gestational age (11/13, 85%). The population was considered to be clearly defined as representative of low-risk pregnancy in a third of studies (4/13; 31%) (Fig 2).

### Risk of stillbirth at term

**Fig 3:**
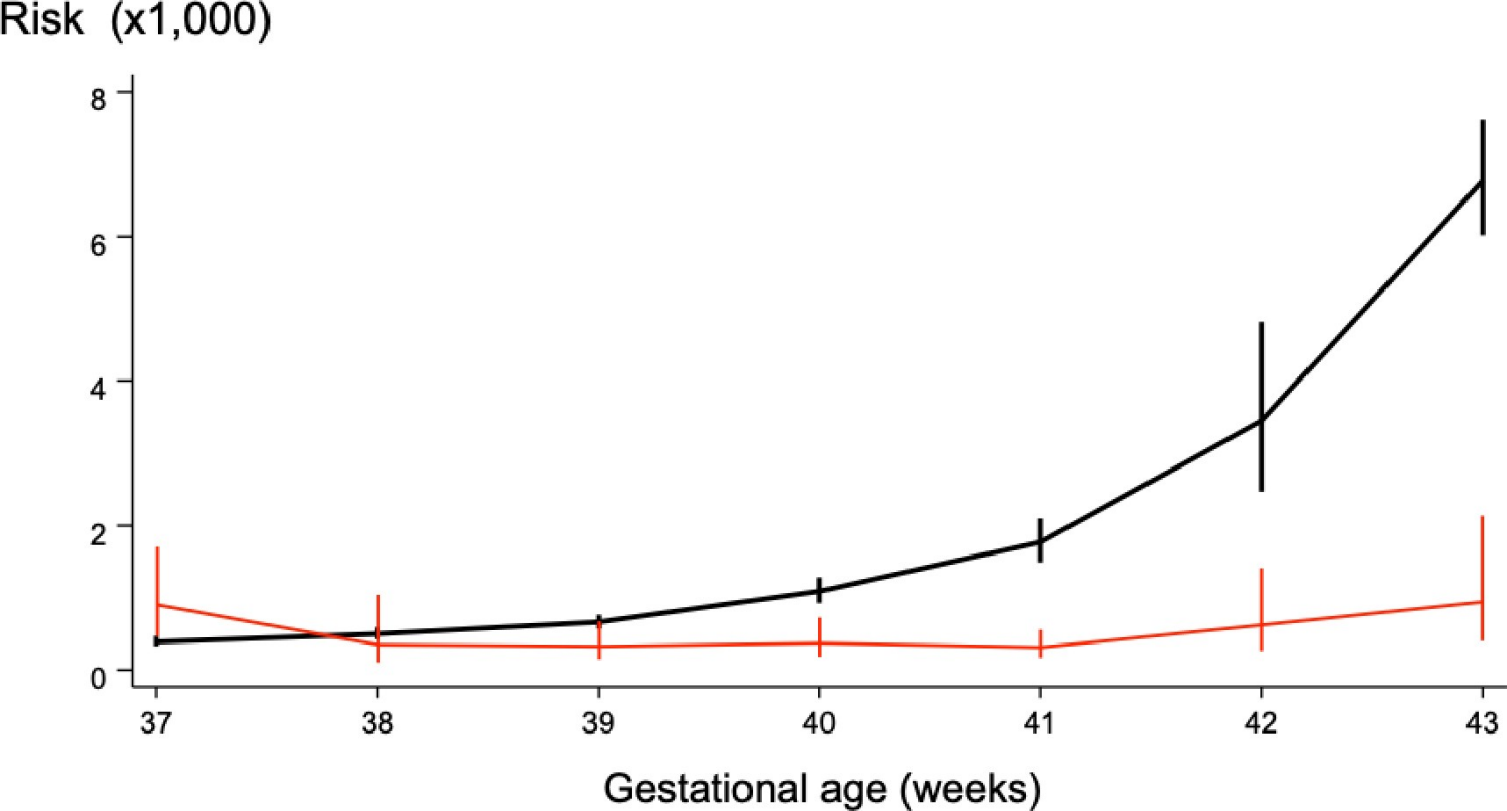
Prospective risk of stillbirth per 1,000 pregnancies and risk of neonatal death per 1,000 deliveries by gestational age in pregnancies continued to term. Stillbirth risk (solid back line); neonatal death risk (solid red line).

The risk of stillbirth at term in the studies varied from 1.1 [34] to 3.2 [12] per 1,000 pregnancies. The overall gestation-week-specific prospective risk of stillbirth steadily increased with gestational age, from 0.11 per 1,000 pregnancies at 37 weeks (95% CI 0.07 to 0.15) to 3.18 per 1,000 at 42 weeks gestation (95% CI 1.84 to 4.35) (Fig 3).

The stillbirth risk increased by 64% (RR 1.64, 95% CI 1.51 to 1.77, *p* < 0.001) when pregnancies are continued to 41 weeks—as currently recommended—compared to delivery at 40 weeks. One additional stillbirth occurred for every 1,449 women (95% CI 1,237 to 1,747) who continued the pregnancy from 40 to 41 weeks (Table 2). S2 Appendix provides individual study estimates on week-specific risks of stillbirth for 40 weeks and 41 weeks.

**Table 2:** Prospective risks of stillbirth and neonatal death for 2 consecutive weeks at term, and the number needed to harm (NNH) for 1 additional stillbirth when pregnancy is continued to the next week.

Gestational age (weeks)	Number of studies	Number of stillbirths	Number of pregnancies	Risk ratio*	95% CI**	Risk difference* (×1,000)	95% CI**	NNH***	95% CI**
**Stillbirth**									
37^+0–6^	12	3,250	8,566,961	1.29	1.18, 1.40	0.11	0.07, 0.15	9,058	6,714, 13,724
38^+0–6^	12	3,516	8,032,865	1.32	1.22, 1.44	0.16	0.11, 0.21	6,242	4,735, 8,839
39^+0–6^	12	3,620	6,784,040	1.64	1.51, 1.79	0.42	0.35, 0.50	2,367	1,997, 2,852
40^+0–6^	12	3,426	4,687,330	1.64	1.51, 1.77	0.69	0.57, 0.81	1,449	1,237, 1,747
41^+0–6^	12	2,407	2,273,471	1.94	1.72, 2.19	1.66	1.29, 2.06	604	486, 775
42^+0–6^	12	1,335	700,610	1.93	1.50, 2.36	3.18	1.84, 4.35	315	230, 543
≥43	6	276	82,039	—	—	—	—	—	—
**Neonatal death**									
37^+0–6^	5	296	552,964	0.41	0.26, 0.57	−0.52	−0.76, −0.31	−1,923	−3,226, −1,316
38^+0–6^	5	428	1,210,730	0.94	0.68, 1.49	−0.02	−0.14, 0.12	−50,000	−7,143, 8,333
39^+0–6^	5	560	2,029,277	1.13	0.90, 1.50	0.05	−0.04, 0.14	20,000	−25,000, 7,143
40^+0–6^	5	669	2,197,643	0.85	0.64, 1.13	−0.06	−0.15, 0.04	−16,667	−6,667, 25,000
41^+0–6^	5	347	1,127,117	1.87	1.07, 2.86	0.28	0.02, 0.54	3,571	1,852, 50,000
42^+0–6^	4	44	70,322	1.32	0.20, 3.38	0.19	−0.52, 1.22	5,263	−1,923, 820
≥43	4	4	6,370	—	—	—	—	—	—

*Risk differences and risk ratios refer to the change in the risk of delivering 1 week later as compared to delivering at that age.

**Bootstrap CI 95% (P_2.5th_, P_97.5th_).

***Number needed to harm when pregnancy is prolonged to the next week, compared to delivery at that gestation, to experience 1 additional stillbirth or neonatal death.

Our sensitivity analyses restricted to studies with a strict definition of low-risk pregnancy (Table 3), pregnancies without congenital fetal malformations (Table 3), last participant recruitment after 1990 (S3 Appendix), and a low risk of bias (S4 Appendix) showed a consistent increase in risk of stillbirth at each gestational week after 37 weeks. Subgroup analyses by race showed that compared to White women, Black women at term were 1.5 to 2 times more likely to have a stillbirth at all gestational ages (S5 Appendix) [12,32,34]. The week-specific prospective risks of stillbirth are provided separately for Black and White women in S6 Appendix. There were no statistically significant differences in the odds of stillbirth at any gestational age between Asian and White women, except for a lower risk at 42 weeks in mothers of Asian race (RR 0.49, 95% CI 0.29 to 0.83, *p* = 0.008) (S7 Appendix) [34].

**Table 3:** Risks of stillbirth in pregnancies that continue to the next week versus deliver in studies with a strict definition of low-risk pregnancy and those without congenital fetal malformations.

Gestational age (weeks)	Number of studies	Number of stillbirths	Number of pregnancies	Risk ratio*	95% CI **	Risk difference* (×1,000)	95% CI **
**Strict definition of low-risk pregnancy**							
37^+0–6^	5	1,297	5,109,474	—	—	—	—
38^+0–6^	5	1,520	4,689,811	1.38	1.18, 1.66	0.12	0.06, 0.20
39^+0–6^	5	1,511	3,763,774	1.33	1.09, 1.66	0.14	0.04, 0.27
40^+0–6^	5	1,266	2,359,848	1.59	1.27, 1.87	0.33	0.19, 0.47
41^+0–6^	5	821	1,009,544	1.88	1.58, 2.31	0.80	0.57, 1.14
42^+0–6^	5	307	243,823	1.52	1.23, 1.80	0.88	0.42, 1.27
≥43	2	13	3212	—	—	—	—
**No congenital fetal malformations**							
37^+0–6^	6	2,156	6,937,382	—	—	—	—
38^+0–6^	6	2,336	6,454,989	1.25	1.12, 1.43	0.08	0.04, 0.13
39^+0–6^	6	2,432	5,368,686	1.39	1.25, 1.56	0.16	0.11, 0.22
40^+0–6^	6	2,237	3,607,608	1.68	1.48, 1.93	0.39	0.29, 0.52
41^+0–6^	6	1,520	1,679,338	1.61	1.39, 1.82	0.58	0.42, 0.75
42^+0–6^	6	739	493,272	1.84	1.58, 2.38	1.29	0.93, 2.07
≥43	3	223	63,934	2.46	1.74, 2.99	4.19	2.80, 5.34

Low-risk pregnancy defined as singleton pregnancies, absence of congenital fetal malformations, and absence of any medical conditions in the mother.

*Between 2 consecutive weeks.

**Bootstrap CI 95% (P_2.5th_, P_97.5th_).

There were insufficient data to undertake other planned subgroup analyses on maternal body mass index, age, and country income status. We did not observe evidence of small study effect for stillbirth (Begg’s test Kendall’s score *p* > 0.05; Egger’s test *p* > 0.05) (S10 Appendix).

### Risk of neonatal mortality at term

The risk of neonatal death was unchanged for births between 38 and 41 weeks of gestation; the risk increased beyond 41 weeks (RR 1.87, 95% CI 1.07 to 2.86, *p* = 0.012). Table 2 provides the estimates of week-specific risk of neonatal death for births at various gestational ages at term. Sensitivity analysis performed by only including studies on singleton pregnancies uncomplicated by congenital fetal malformations (S8 Appendix), and only high-quality studies (S9 Appendix), showed a similar pattern, with increased risks observed for births beyond 42 weeks compared to the previous week.

## Discussion

We found that the prospective risk of stillbirth increased with gestational age in pregnancies at term; neonatal mortality risk remained unchanged until 41 weeks, but increased beyond this gestation. Pregnancies that continued to 41 weeks—currently still considered normal term gestation—had a small but significant increase in the risk of stillbirth compared to those delivered at 40 weeks, with no differences in neonatal mortality.

To our knowledge, ours is the largest review to date on risks of stillbirth and neonatal death at various gestational ages in term pregnancies. The review was based on a prospective protocol with predefined inclusion criteria. We registered the review protocol with PROSPERO prior to completion of the detailed search and data extraction. We contacted the individual authors for relevant data when it was required for the analysis and where it was possible. When we included unpublished data, the relevant researchers were not involved in the data extraction, quality assessment, or analysis to minimise bias. The large sample size achieved with these efforts allowed us to generate results with high precision. By reporting both relative and absolute increases in the risks, our findings provide the appropriate context for interpretation. We assessed the qualities of the included studies and the validity of the evidence. Our sensitivity analyses demonstrated that our findings were not sensitive to the assumptions made. Unlike previous studies in this area, our robust analytical approach [38,39] avoided the inappropriate use of Kaplan–Meier method [40,41].

The inclusion criteria varied between studies. But all studies included women whose pregnancy continued to term and beyond, an indication of their low-risk status in that early delivery was not required [42,43]. Some of these apparently ‘low risk’ pregnancies may also have had undetected fetal growth restriction. But continuation of such pregnancies to term is in line with current practice, where there is no routine ultrasound monitoring of fetal growth [43].

Some of the included cohorts extended before 1990, and the risk of outcomes might have changed over time. We consider the effect of study time span on our stillbirth estimates to be minimal for the following reasons. First, current definitions of ‘term’ and ‘post-term’ pregnancies have remained unchanged over the decades, with very little change in the criteria that label pregnancies as high or low risk [44,45]. Second, evidence behind the current recommendations on the timing of delivery in term pregnancies with no obvious reasons for early delivery stem from both recent and past data [6,46] Third, in apparently low-risk pregnancies at term, the standard of antenatal care, including regular blood pressure checks and auscultation of fetal heart, has not changed over time [44]. Lastly, we observed very little between-study variance in the reported risks of stillbirth for various gestational ages, irrespective of the year of data collection or inclusion criteria.

Although the database registries are prone to biases, we expect the outcome of death to be well recorded [47]. It is possible that an intrauterine death recorded as being in a particular gestational week may have occurred in the previous week. But in women who undergo weekly monitoring of fetal heart rate at term gestation, the time interval from fetal demise to birth is considered about 2 days on average [48].

Regarding maternal characteristics, we only analysed the risk of stillbirth at various gestational ages by race. Due to the paucity of the published information, we were unable to explore in detail if there were variations in risks by socioeconomic status, maternal age, and parity [49]. Our approach is similar to that of previous studies that studied the ‘real life’ risk of stillbirth with advancing gestation at term irrespective of the presence or absence of risk factors [30]. The higher risk of stillbirth at all gestational ages in Black women compared to White women could be attributed to upstream determinants such as low educational and socioeconomic status, reduced access to antenatal care, and increased rates of fetal growth restriction [50–52].

We did not observe significant changes in neonatal mortality for births between 38 and 41 weeks gestation, a finding that was consistent with previous studies [53,54]. The developmental outcomes for children born at term but before 40 weeks gestation are not known to be significantly different from those for children born at 40 weeks [55]. In a recently published randomised trial (ARRIVE) on induction versus expectant management in low-risk nulliparous women, the reduction in the composite of perinatal death or serious neonatal complication by 20% with induction fell just short of statistical significance (95% CI 0.64 to 1.00) [56]; our findings also support the evidence of benefit for delivery versus expectant management. Trials and systematic reviews have assessed the effects of induction of labour at prespecified gestational cutoffs such as 39 weeks [57,58]. This approach limits the information on risks of stillbirth at various gestational time points, and the effects of intervention.

Any discussion with women considering prolonging their pregnancy beyond 41 weeks gestation should be include information on the absolute risk increase, and the effects of induction of labour on mode of delivery and perinatal outcomes [58]. There is a need to assess the acceptability of early delivery at term to parents and healthcare providers to avoid the small risk of stillbirth. Better stratification of apparently low-risk pregnancies for complications using individualised prediction models could reduce the number of women who need to be delivered to avoid 1 additional stillbirth. Decision analytic modelling with economic evaluation is required to assess the cost-effectiveness of offering delivery at various gestational ages at term.

While our comprehensive systematic review provided robust quantitative estimates of the risks of stillbirth and neonatal death at various gestational ages in term pregnancies, the findings were limited by the heterogeneity in the definition of low-risk pregnancies (which might have included women with undiagnosed fetal growth restriction), loss of data due to exclusion of studies that did not provide stillbirth estimates in weekly intervals, and the inability to adjust for confounding variables.

In conclusion, there is a significant increase in the risk of stillbirth, without a corresponding reduction in the risk of neonatal death, in mothers at term when pregnancies continue to the current recommended gestation of 41 weeks compared to delivering in the previous week.

## Supporting information

S1 Appendix Search strategies. (DOCX)

S2 Appendix Individual study estimates for prospective risks of stillbirths at 40 weeks and 41 weeks in women at term gestation. (DOCX)

S3 Appendix Risk ratios and risk differences for stillbirth when term pregnancy is continued to the next week versus delivery at various gestational ages in studies where last participant was recruited after 1990. (DOCX)

S4 Appendix Risk ratio and risk difference for stillbirth and neonatal death when pregnancies continue versus deliver at various gestational ages in studies with low risk of bias. (DOCX)

S5 Appendix Prospective weekly risks of stillbirth in pregnancies continued to 37 weeks and beyond in Black compared to White mothers. (DOCX)

S6 Appendix Prospective risks of stillbirth in pregnancies that are continued versus delivered at various gestational ages at term, by race. (DOCX)

S7 Appendix Risks of stillbirth at various gestational ages in Asian versus White mothers at term. (DOCX)

S8 Appendix Risks of neonatal death in pregnancies that continue to the next week versus delivery in studies on singleton pregnancies without congenital fetal malformations. (DOCX)

S9 Appendix Risk ratio and risk difference of neonatal death when pregnancies continue versus deliver at various gestational ages in studies with low risk of bias. (DOCX)

S10 Appendix Publication bias and small study effect amongst studies included in the systematic review and meta-analysis of stillbirth risk in term pregnancies. (DOCX)

S1 PRISMA checklist (DOCX)

## Abbreviations

OR: odds ratio
RR: risk ratio
