# Supplementary material for: Risks of stillbirth and neonatal death with advancing gestation at term: A systematic review and meta-analysis of cohort studies of 15 million pregnancies
Source: PLoS Med. 2019 Jul 2;16(7):e1002838. doi: 10.1371/journal.pmed.1002838 (PMC6605635; doi:10.1371/journal.pmed.1002838)
Supplement: S1 Appendix — (DOCX) [file pmed.1002838.s001.docx]

**S1 Appendix: Search strategies in Medline for the systematic review for stillbirths and neonatal deaths in pregnancies continued to term gestation: A meta-analysis of 15 million pregnancies**

a) Search strategy for studies on stillbirth, neonatal and perinatal death

1. (singleton pregnancy).ti.ab
2. (prolonged pregnancy) .ti.ab
3. (term pregnancy).ti.ab
4. (low-risk pregnancy).ti.ab
5. (post-term pregnancy).ti.ab
6. (gestational age). ti,ab
7. (post-dates pregnancy). ti,ab
8. exp PROLONGED PREGNANCY
9. exp TERM BIRTH
10. exp GESTATIONAL AGE
11. (stillbirth). ti, ab
12. (perinatal death). ti, ab
13. (peri-natal death). ti, ab
14. (neonatal death). ti, ab
15. (intrauterine death). ti, ab
16. (intra-uterine death). ti, ab
17. (IUD). ti, ab
18. (newborn mortality). ti, ab
19. exp STILLBIRTH
20. exp FETUS DEATH
21. exp PERINATAL DEATH
22. 1 OR 2 OR 3 OR 4 OR 5 OR 6 OR 7 OR 8 OR 9 OR 10
23. 11 OR 12 OR 13 OR 14 OR 15 OR 16 OR 17 OR 18 OR 19 OR 20 OR 21
24. 22 AND 23

b) Additional search for studies on only neonatal and/or perinatal death

1. (singleton AND pregnan*).ti,ab;
2. (term AND pregnan*).ti,ab
3. low-risk AND pregnan*).ti,ab
4. 1 OR 2 OR 3
5. (neonat* AND death).ti,ab
6. neonat* AND mortality).ti,ab
7. (neonat* AND outcome).ti,ab
8. (neonat* AND morbidity).ti,ab
9. exp PERINATAL DEATH
10. 5 OR 6 OR 7 OR 8 OR 9
11. 4 AND 10
