# Supplementary material for: Risks of stillbirth and neonatal death with advancing gestation at term: A systematic review and meta-analysis of cohort studies of 15 million pregnancies
Source: PLoS Med. 2019 Jul 2;16(7):e1002838. doi: 10.1371/journal.pmed.1002838 (PMC6605635; doi:10.1371/journal.pmed.1002838)

**S2 Appendix: Individual study estimates for prospective risks of stillbirths at 40 weeks and 41 weeks in women at term gestation.**

1. Rates of stillbirth at 40 weeks of gestation


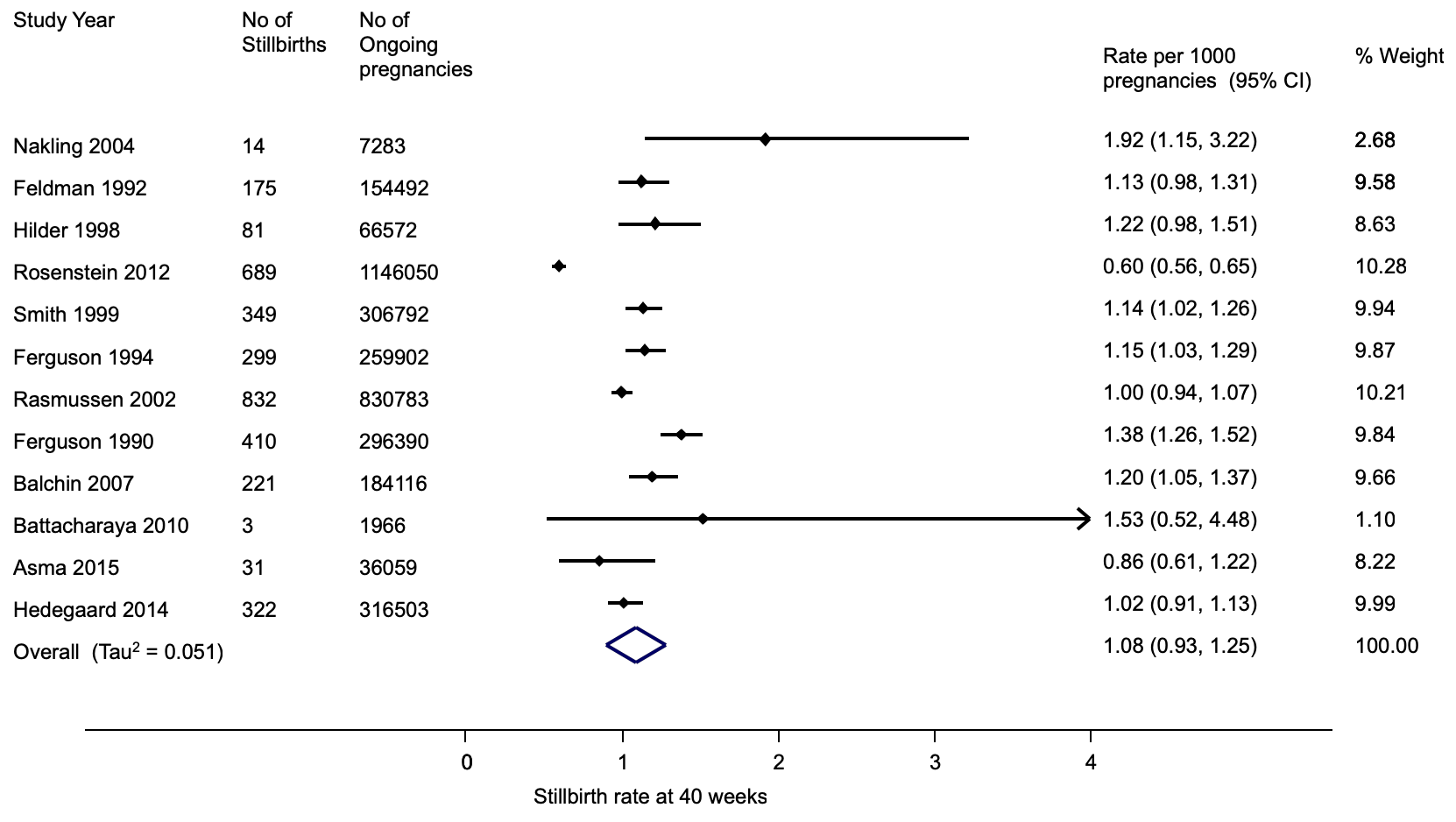


1. Rates of stillbirth at 41 weeks of gestation


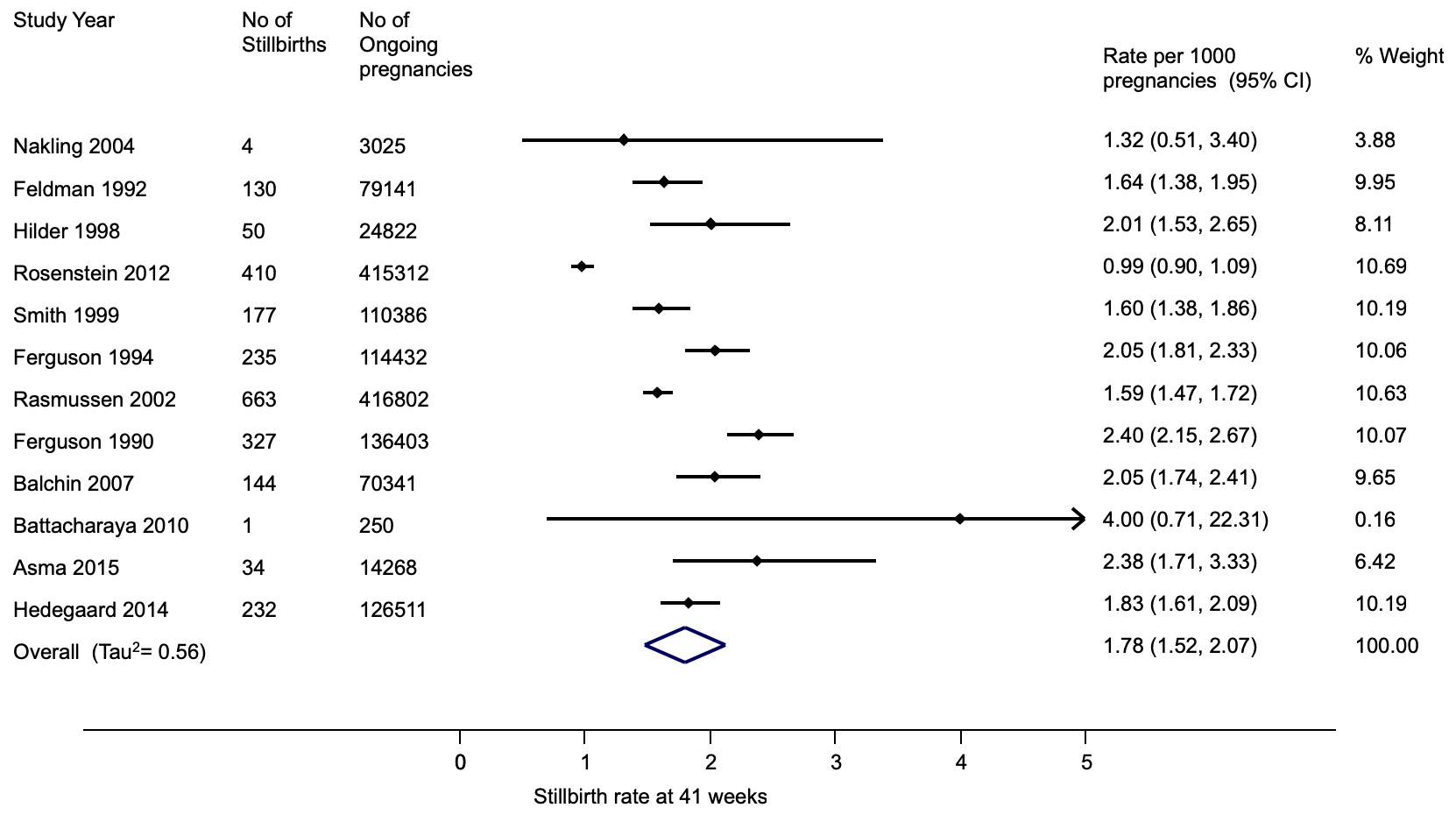

Supplement: S2 Appendix — (DOCX) [file pmed.1002838.s002.docx]
