# Supplementary material for: Risks of stillbirth and neonatal death with advancing gestation at term: A systematic review and meta-analysis of cohort studies of 15 million pregnancies
Source: PLoS Med. 2019 Jul 2;16(7):e1002838. doi: 10.1371/journal.pmed.1002838 (PMC6605635; doi:10.1371/journal.pmed.1002838)
Supplement: S3 Appendix — (DOCX) [file pmed.1002838.s003.docx]

**S3 Appendix: Risk ratios and risk differences for stillbirth when term pregnancy is continued to the next week vs. delivery at various gestational ages. Data limited to studies where last participant was recruited after 1990**

*Between two consecutive weeks

^**^ Bootstrap CI 95% (P_2.5th_, P_97.5th_)

| **Gestational age**  **(weeks)** | **No. of studies** | **No. of stillbirths** | **No. of pregnancies** | **Risk ratio^*^** | **(95% CI) ^**^** | **Risk difference^*^ (x1,000)** | **(95% CI) ^**^** |
| --- | --- | --- | --- | --- | --- | --- | --- |
| 37^+0-6^ | 9 | 2397 | 7,581,551 | 1.35 | (1.22, 1.50) | 0.12 | (0.08, 0.16) |
| 38^+0-6^ | 9 | 2655 | 7,055,895 | 1.30 | (1.18, 1.52) | 0.14 | (0.09, 0.23) |
| 39^+0-6^ | 9 | 2714 | 5,869,655 | 1.69 | (1.47, 1.91) | 0.42 | (0.31, 0.53) |
| 40^+0-6^ | 9 | 2542 | 3,956,311 | 1.63 | (1.44, 1.85) | 0.64 | (0.49, 0.82) |
| 41^+0 -6^ | 9 | 1715 | 1,835,944 | 1.65 | (1.43, 1.96) | 1.08 | (0.76, 1.57) |
| 42^+0-6^ | 9 | 788 | 527,494 | 2.55 | (1.85, 3.09) | 4.31 | (2.45, 5.57) |
| ≥43 | 5 | 240 | 68,550 | - | - | - | - |
