# Supplementary material for: Risks of stillbirth and neonatal death with advancing gestation at term: A systematic review and meta-analysis of cohort studies of 15 million pregnancies
Source: PLoS Med. 2019 Jul 2;16(7):e1002838. doi: 10.1371/journal.pmed.1002838 (PMC6605635; doi:10.1371/journal.pmed.1002838)
Supplement: S4 Appendix — (DOCX) [file pmed.1002838.s004.docx]

**S4 Appendix: Risk ratio and risk difference for stillbirth and neonatal death when pregnancies continue vs. deliver at various gestational ages in studies with low risk of bias**

**a. Stillbirth**

| **Gestational age**  **(weeks)** | **No. of studies** | **No. of stillbirths** | **No. of pregnancies** | **Risk ratio^*^** | **(95% CI) ^**^** | **Risk difference^*^ (x1,000)** | **(95% CI) ^**^** |
| --- | --- | --- | --- | --- | --- | --- | --- |
| 37^+0-6^ | 5 | 1,297 | 5,109,474 | 1.39 | (1.18; 1.65) | 0.12 | (0.06; 0.19) |
| 38^+0-6^ | 5 | 1,520 | 4,689,811 | 1.33 | (1.09; 1.68) | 0.14 | (0.04; 0.28) |
| 39^+0-6^ | 5 | 1,511 | 3,763,774 | 1.60 | (1.29; 1.89) | 0.34 | (0.19; 0.49) |
| 40^+0-6^ | 5 | 1,266 | 2,359,848 | 1.89 | (1.58; 2.39) | 0.80 | (0.58; 1.22) |
| 41^+0 -6^ | 5 | 821 | 1,009,544 | 1.30 | (1.05; 1.57) | 0.52 | (0.09; 0.94) |
| 42^+0-6^ | 5 | 307 | 243,823 | NC | NC | NC | NC |
| ≥43 | 2 | 13 | 3,212 | - | - | - | - |

*Between two consecutive weeks

^**^ Bootstrap CI 95% (P_2.5th_, P_97.5th_)

NC: Not convergence of the model

**b. Neonatal death**

| **Gestational age**  **(weeks)** | **No. of studies** | **No. of neonatal deaths** | **No. of deliveries** | **Risk ratio^*^** | **(95% CI) ^**^** | **Risk difference^*^ (x1,000)** | **(95% CI) ^**^** |
| --- | --- | --- | --- | --- | --- | --- | --- |
| 37^+0-6^ | 2 | 21 | 39,338 | 0·16 | (0·05; 0·43) | -0·52 | (-0·85; -0·21) |
| 38^+0-6^ | 2 | 8 | 89,223 | 1·58 | (0·67; 5·13) | 0·06 | (-0·05; 0·17) |
| 39^+0-6^ | 2 | 21 | 139,183 | 1·24 | (0·67; 2·4) | 0·04 | (-0·07; 0·15) |
| 40^+0-6^ | 2 | 31 | 160,289 | 0·83 | (0·4; 1·51) | -0·03 | (-0·16; 0·08) |
| 41^+0 -6^ | 2 | 17 | 110,843 | 1·97 | (0·47; 5·26) | 0·16 | (-0·09; 0·49) |
| 42^+0-6^ | 2 | 8 | 25,976 | 1·79 | (0·52; 9·72) | 0·24 | (-0·27; 1·59) |
| ≥43 | 2 | 2 | 3,212 | - | - | - | - |

*Between two consecutive weeks

^**^ Bootstrap CI 95% (P_2.5th_, P_97.5th_)
