# Supplementary material for: Risks of stillbirth and neonatal death with advancing gestation at term: A systematic review and meta-analysis of cohort studies of 15 million pregnancies
Source: PLoS Med. 2019 Jul 2;16(7):e1002838. doi: 10.1371/journal.pmed.1002838 (PMC6605635; doi:10.1371/journal.pmed.1002838)
Supplement: S5 Appendix — (DOCX) [file pmed.1002838.s005.docx]

**S5 Appendix: Prospective weekly risks of stillbirths in pregnancies continued to 37 weeks and beyond in Black compared to White mothers**
