# Supplementary material for: Risks of stillbirth and neonatal death with advancing gestation at term: A systematic review and meta-analysis of cohort studies of 15 million pregnancies
Source: PLoS Med. 2019 Jul 2;16(7):e1002838. doi: 10.1371/journal.pmed.1002838 (PMC6605635; doi:10.1371/journal.pmed.1002838)
Supplement: S6 Appendix — (DOCX) [file pmed.1002838.s006.docx]

**S6 Appendix: Prospective risks of stillbirths in pregnancies that are continued vs. delivered at various gestational ages at term by ethnicity**

a. White women

| **Gestational age**  **(weeks)** | **No. of studies** | **No. of stillbirths** | **No. of pregnancies** | **Risk ratio^*^** | **(95% CI) ^**^** | **Risk difference^*^ (x1,000)** | **(95% CI) ^**^** | **Number at risk of one additional stillbirth ^***^** | **(95% CI) ^**^** |
| --- | --- | --- | --- | --- | --- | --- | --- | --- | --- |
| 37^+0-6^ | 5 | 1,038 | 3,354,963 | 1,06 | (0,88; 1,22) | 0,02 | (-0,04; 0,07) | 46,632 | (14,158; -25,598) |
| 38^+0-6^ | 5 | 1,104 | 3,114,895 | 1,57 | (1,36; 1,92) | 0,20 | (0,14; 0,28) | 4,883 | (3,512; 7,213) |
| 39^+0-6^ | 5 | 1,201 | 2,578,106 | 1,60 | (1,43; 1,82) | 0,34 | (0,25; 0,44) | 2,947 | (2,291; 3,937) |
| 40^+0-6^ | 5 | 1,084 | 1,708,129 | 1,84 | (1,59; 2,09) | 0,75 | (0,57; 0,94) | 1,325 | (1,059; 1,753) |
| 41^+0 -6^ | 5 | 827 | 798,495 | 2,38 | (1,95; 2,85) | 2,29 | (1,60; 2,89) | 437 | (346; 624) |
| 42^+0-6^ | 5 | 545 | 220,913 | - | - | - | - | - | - |

b. Black women

| **Gestational age**  **(weeks)** | **No. of studies** | **No. of neonatal deaths** | **No. of deliveries** | **Risk ratio^*^** | **(95% CI) ^**^** | **Risk difference^*^ (x1,000)** | **(95% CI) ^**^** | **Number at risk of one additional stillbirth ^***^** | **(95% CI) ^**^** |
| --- | --- | --- | --- | --- | --- | --- | --- | --- | --- |
| 37^+0-6^ | 2 | 297 | 499,379 | 1·37 | (1·09; 1·74) | 0·21 | (0·06; 0·36) | 4,764 | (2,809; 17,083) |
| 38^+0-6^ | 2 | 319 | 445,508 | 1·20 | (0·89; 1·49) | 0·15 | (-0·09; 0·33) | 6,681 | (3,030; -10,845) |
| 39^+0-6^ | 2 | 290 | 351,529 | 1·44 | (1·07; 1·94) | 0·41 | (0·07; 0·70) | 2,449 | (1,419; 14,465) |
| 40^+0-6^ | 2 | 244 | 220,757 | 1·68 | (1·23; 2·23) | 0·89 | (0·34; 1·39) | 1,126 | (721; 2,923) |
| 41^+0 -6^ | 2 | 163 | 102,657 | 2·42 | (1·70; 3·48) | 3·16 | (1·68; 4·70) | 317 | (213; 597) |
| 42^+0-6^ | 2 | 103 | 32,007 | - | - | - | - | - | - |

* Risk difference and risk ratio refers to the change in the risk of delivering one week later as compared to delivering at that age.

^**^ Bootstrap CI 95% (P_2.5th_, P_97.5th_)

^***^ Number at risk when pregnancy is prolonged to the next week, compared to delivery at that gestation, to experience one additional stillbirth.
