# Supplementary material for: Risks of stillbirth and neonatal death with advancing gestation at term: A systematic review and meta-analysis of cohort studies of 15 million pregnancies
Source: PLoS Med. 2019 Jul 2;16(7):e1002838. doi: 10.1371/journal.pmed.1002838 (PMC6605635; doi:10.1371/journal.pmed.1002838)
Supplement: S7 Appendix — (DOCX) [file pmed.1002838.s007.docx]

**S7 Appendix: Risks of stillbirth at various gestational ages in Asian vs. White mothers at term**

| n = 2 studies | | | | | | | | | |
| --- | --- | --- | --- | --- | --- | --- | --- | --- | --- |
| Gestational age (weeks) | White women | | | Asian women | | | Asian vs White  (Ref. White) | Heterogeneity | |
|  | No. of stillbirths | No. of ongoing pregnancies | Risk of stillbirth (95% CI)  (x1000) | No. of stillbirths | No. of ongoing  pregnancies | Risk of stillbirth (95% CI) (x1000) | OR  (95% CI) | Tau  squared |  |
| 37^+0 -6^ | 756 | 2,582,480 | 0·33 (0·23, 0·47) | 92 | 449,269 | 0·21 (0·18, 0·26) | 0·79 (0·33, 1·89) | 0·00 |  |
| 38^+0 -6^ | 798 | 2,390,755 | 0·33 (0·21, 0·53) | 105 | 407,304 | 0·29 (0·24, 0·35) | 0·98 (0·79, 1·22) | 0·00 |  |
| 39^+0 -6^ | 874 | 1,969,454 | 0·55 (0·39, 0·76) | 90 | 313,398 | 0·37 (0·30, 0·45) | 0·72 (0·46, 1·13) | 0·00 |  |
| 40^+0 -6^ | 762 | 1,278,835 | 0·88 (0·61, 1·26) | 79 | 177,241 | 0·65 (0·52, 0·81) | 0·72 (0·36, 1·44) | 0·00 |  |
| 41^+0 -6^ | 595 | 594,782 | 1·62 (1·09, 2·42) | 52 | 67,628 | 2·00 (0·71, 5·66) | 0·89 (0·51, 1·57) | 0·53 |  |
| 42^+0 -6^ | 457 | 171,603 | 4·04 (2·08, 7·86) | 17 | 16,394 | 2·06 (1·28, 3·32) | 0·49 (0·29, 0·83) | 0·00 |  |
