# Supplementary material for: Risks of stillbirth and neonatal death with advancing gestation at term: A systematic review and meta-analysis of cohort studies of 15 million pregnancies
Source: PLoS Med. 2019 Jul 2;16(7):e1002838. doi: 10.1371/journal.pmed.1002838 (PMC6605635; doi:10.1371/journal.pmed.1002838)
Supplement: S8 Appendix — (DOCX) [file pmed.1002838.s008.docx]

**S8 Appendix: Risks of neonatal death in pregnancies that continue to the next week vs. delivery in studies on singleton pregnancies without congenital fetal abnormalities**

| **Gestational age**  **(weeks)** | **No. of studies** | **No. of**  **neonatal**  **deaths** | **No. of births** | **Risk ratio^*^** | **(95% CI) ^**^** | **Risk difference^*^ (x1,000)** | **(95% CI) ^**^** |
| --- | --- | --- | --- | --- | --- | --- | --- |
| 37^+0-6^ | 3 | 261 | 510,644 | - | - | - | - |
| 38^+0-6^ | 3 | 380 | 1,112,842 | 0·48 | (0·33, 0·66) | -0·44 | (-0·69, -0·21) |
| 39^+0-6^ | 3 | 504 | 1,880,213 | 0·80 | (0·61, 1·04) | -0·08 | (-0·18, 0·02) |
| 40^+0-6^ | 3 | 580 | 2,008,479 | 1·10 | (0·81, 1·45) | 0·03 | (-0·07, 0·12) |
| 41^+0 -6^ | 3 | 314 | 1,005,232 | 1·01 | (0·72, 1·37) | 0·01 | (-0·11, 0·10) |
| 42^+0-6^ | 2 | 25 | 40,717 | 1·79 | (1·02, 2·86) | 0·28 | (0·01, 0·59) |
| ≥43 | 2 | 1 | 1,754 | 1·79 | (0·68, 5·33) | 0·50 | (-0·29, 2·30) |

*Between two consecutive weeks

^**^ Bootstrap CI 95% (P_2.5th_, P_97.5th_)
