# Supplementary material for: Risks of stillbirth and neonatal death with advancing gestation at term: A systematic review and meta-analysis of cohort studies of 15 million pregnancies
Source: PLoS Med. 2019 Jul 2;16(7):e1002838. doi: 10.1371/journal.pmed.1002838 (PMC6605635; doi:10.1371/journal.pmed.1002838)

**S10 Appendix: Publication bias and small study effect amongst studies included in the systematic review and meta-analysis on stillbirth risk in term pregnancies**


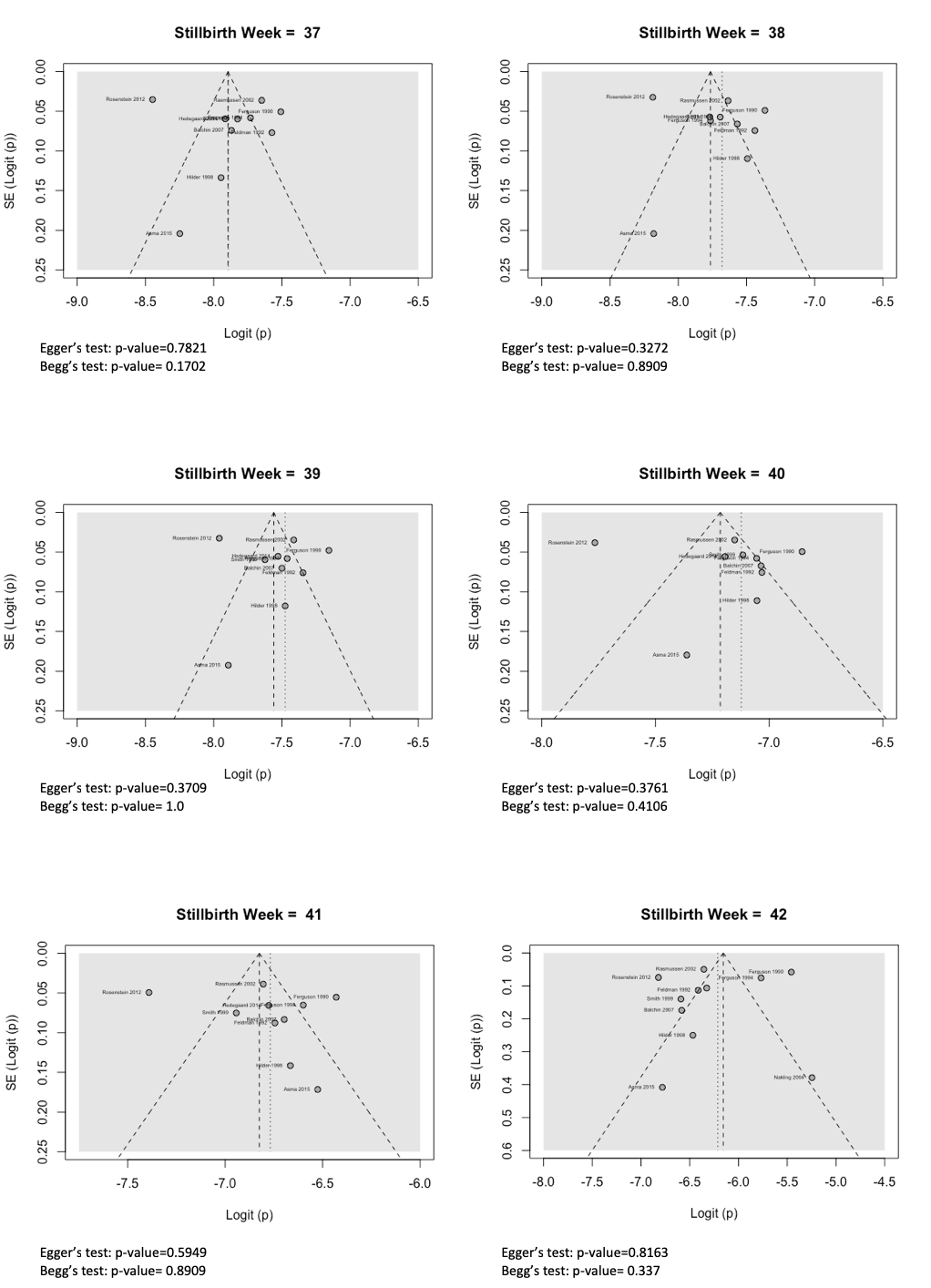

Supplement: S10 Appendix — (DOCX) [file pmed.1002838.s010.docx]
